# Ubiquitin ligase RNF125 targets PD-L1 for ubiquitination and degradation

**DOI:** 10.3389/fonc.2022.835603

**Published:** 2022-07-29

**Authors:** Meng Wei, Yunhai Mo, Jialong Liu, Jingtong Zhai, Huilong Li, Yixin Xu, Yumeng Peng, Zhihong Tang, Tao Wei, Xiaopan Yang, Linfei Huang, Xiao Shao, Jingfei Li, Li Zhou, Hui Zhong, Congwen Wei, Qiaosheng Xie, Min Min, Feixiang Wu

**Affiliations:** ^1^ Department of Hepatobiliary Surgery, Affiliated Tumor Hospital of Guangxi Medical University, Nanning, China; ^2^ Key Laboratory of High-Incidence-Tumor Prevention and Treatment (Guangxi Medical University), Ministry of Education, Nanning, China; ^3^ Department of Genetic Engineering, Beijing Institute of Biotechnology, Beijing, China; ^4^ Department of Medical Oncology and State Key Laboratory of Molecular Oncology, National Cancer Center/ Cancer Hospital, Chinese Academy of Medical Sciences and Peking Union Medical College, Beijing, China; ^5^ Department of Gastroenterology, First Medical Center of Chinese People's Liberation Army General Hospital, Beijing, China; ^6^ Department of Radiation Oncology, China-Japan Friendship Hospital, Beijing, China

**Keywords:** RNF125, PD-L1, ubiquitination, tumor immunotherapy, clinical outcome

## Abstract

As a critical immune checkpoint molecule, PD-L1 is expressed at significantly higher levels in multiple neoplastic tissues compared to normal ones. PD-L1/PD-1 axis is a critical target for tumor immunotherapy, blocking the PD-L1/PD-1 axis is recognized and has achieved unprecedented success in clinical applications. However, the clinical efficacy of therapies targeting the PD-1/PD-L1 pathway remains limited, emphasizing the need for the mechanistic elucidation of PD-1/PD-L1 expression. In this study, we found that RNF125 interacted with PD-L1 and regulated PD-L1 protein expression. Mechanistically, RNF125 promoted K48-linked polyubiquitination of PD-L1 and mediated its degradation. Notably, MC-38 and H22 cell lines with RNF125 knockout, transplanted in C57BL/6 mice, exhibited a higher PD-L1 level and faster tumor growth than their parental cell lines. In contrast, overexpression of RNF125 in MC-38 and H22 cells had the opposite effect, resulting in lower PD-L1 levels and delayed tumor growth compared with parental cell lines. In addition, immunohistochemical analysis of MC-38 tumors with RNF125 overexpression showed significantly increased infiltration of CD4+, CD8+ T cells and macrophages. Consistent with these findings, analyses using The Cancer Genome Atlas (TCGA) public database revealed a positive correlation of RNF125 expression with CD4+, CD8+ T cell and macrophage tumor infiltration. Moreover, RNF125 expression was significantly downregulated in several human cancer tissues, and was negatively correlated with the clinical stage of these tumors, and patients with higher RNF125 expression had better clinical outcomes. Our findings identify a novel mechanism for regulating PD-L1 expression and may provide a new strategy to increase the efficacy of immunotherapy.

## Introduction

The pathogenesis of cancer is complicated and involves multiple driver and passenger genes. Cancer immune escape is considered as a key mechanism by which cancer avoids immune destruction and develops noninheritable resistance to antineoplastic medication (1). During cancer immunoediting, cancer cells tend to evade immune surveillance by manipulating immune checkpoint molecules (2–4). The expression of programmed death ligand-1 (PD-L1), a critical immune checkpoint molecule, was lower in normal tissues than in cancer tissues (5). The engagement of PD-L1 with programmed cell death protein-1 (PD-1) on tumor-infiltrating cytolytic T cells (CTLs) induces inhibitory signaling and impairs their antitumor activity (6). Blocking the PD-L1/PD-1 axis has been approved for treating human cancers and clinical application of blocking PD-1/PD-L1 antibodies against many cancers has shown significant clinical benefits (7, 8). However, some cancer patients lose response to anti-PD-1/PD-L1 treatment, and some of the well-responsive patients showing extraordinary treatment outcomes still relapse (9, 10). Recent studies have suggested that the response to PD-1/PD-L1 inhibitors may be related to the level of PD-L1 in tumors, further emphasizing the need for the mechanistic elucidation of PD-L1 expression regulation (11, 12).

Several lines of evidence suggest that ubiquitination is critical for PD-L1 expression regulation and PD-L1-mediated immune checkpoint signaling pathways. For example, TNF-α secreted under inflammatory states can promote the expression of the deubiquitinating enzyme CSN5, which inhibits the ubiquitination and degradation of PD-L1. Inhibition of CSN5 by curcumin decreases the expression of PD-L1 on tumor cells and renders them more responsive to CTLA4-mediated immunotherapy (13). CMTM6, positioned on the cell surface, can reduce the ubiquitination of the PD-L1 protein, thereby extend the half-life of PD-L1 *in vivo*, and further weakens the tumor-killing ability of T cells. Another CMTM family protein, CMTM4, also regulates PD-L1 turnover by ubiquitination modification (14, 15). Recent studies find that the E3 ligase SPOP can be regulated by CyclinD, CDK4, and Cullin-3 to control the expression of PD-L1. SPOP with loss-of-function mutations cannot ubiquitinate and degrade PD-L1, resulting in increased PD-L1 protein level (16). However, the regulatory mechanisms underlying PD-L1 expression remain, for the most part, elusive.

This study identified the E3 ligase RNF125 as the direct E3 ligase of PD-L1. The E3 ligase activity of RNF125 is indispensable for PD-L1 ubiquitination and proteasome degradation. The inhibitory role of RNF125 on PD-L1 expression determines tumor growth. Thus, this new molecular mechanism that can regulate the stability of PD-L1 protein may be a new target to improve the therapeutic efficacy of immunotherapy against human cancers.

## Materials and methods

### Reagents

Propidium iodide solution (PI; B316099) was purchased from BioLegend (CA, USA). Lymphocyte Separation Medium (P8620) and TriQuick Reagent (R1100) were purchased from Solarbio (Beijing, China). The EasySep™ Mouse CD8+ T Cell Isolation Kit (19753) and EasySep™ Magnet (18000) were purchased from StemCell Technologies (Vancouver, Canada). The Annexin V-FITC Apoptosis Detection Kit (C1062L) and Red Blood Cell Lysis Buffer (C3702) were purchased from Beyotime (Shanghai, China). Pitstop-2 (ab120687) was purchased from Abcam (Illinois, USA). Hydroxy-Dynasore (HY-13863) was purchased from MedChemExpress (New Jersey, USA). CellTiter-Glo^®^ Luminescent Cell Viability Assay (G7570) was purchased from Promega (WI, USA). PerfectStartTM Green qPCR SuperMix (AQ601) and TransScript^®^ one-step gDNA removal and cDNA synthesis Supermix (at311) were purchased from TransGen Biotech (Beijing, China). The transfection reagent jetPRIME (25Y1801N5) and jetPRIME buffer (B210217) were purchased from Polyplus Transfection (Illkirch Graffenstaden, France).

### Antibodies

Anti-Mo CD3e (2074540; 1:100 dilution) and anti-Mo CD8a (2075802; 1:100 dilution) antibodies were purchased from Invitrogen (CA, USA). Alexa Fluor^®^ 647 (B319823; 1:100 dilution) was purchased from BioLegend (CA, USA). Anti-CD8 (ab4055; 1:500 dilution) was purchased from Abcam (Illinois, USA). Anti-RNF125 (A15166; 1:1,000 dilution) was purchased from ABclonal (Wuhan, China). Anti-α-tubulin antibody (T6074; 1:5000 dilution) and anti-Flag (A8592; 1:5000 dilution) antibody were purchased from Sigma–Aldrich (Missouri, USA). Anti-PD-L1 (17952-1-AP; 1:1,000 dilution) and anti-Myc (16286-1-AP; 1:1,000 dilution) antibodies were purchased from Proteintech (Wuhan, China). Anti-HA (14031S; 1:1,000 dilution) was purchased from Cell Signaling Technology (Danvers, USA). Anti-rabbit HRP-IgG (ZB-2301; 1:5000 dilution) and anti-mouse HRP-IgG (ZB-2305; 1:5000 dilution) secondary antibodies were purchased from ZSGB-BIO (Beijing, China). CD4 (GB11064; 1:1000 dilution), CD8 (GB114196; 1:1000 dilution) and F4/80 (GB113373; 1:1000 dilution) antibodies were purchased from Servicebio (WuHan, China).

### Plasmids and siRNA

The corresponding PCR-amplified fragment was inserted into pcDNA3 (Invitrogen) to construct a mammalian expression vector encoding N-terminal Flag-, HA- and Myc-tagged RNF125. All the constructs were verified by DNA sequencing. The target sequence of siRNA for human RNF125-3 was 5’ - CCGGUCACUUCUUGAAUAUTT - 3’. Scrambled siRNA oligonucleotides were used as a control (siCtrl).

### Lentiviral production for over-expression and knockout of related gene

Cell lines with stable overexpression of RNF125 genes were generated as follows. HEK293T cells were transfected with pLVX-empty vector constructs, together with psPAX.2 and pMD2.G third-generation lentiviral packaging system using Lipofectamine 2000 reagent (Life Technologies) according to the manufacturer’s instructions. 72 hours later, lentivirus particles in the medium were collected and filtered, then the target cell lines were infected. At 24 hours post-infection, puromycin was added to obtain stable cell lines with successful transduction. The construction method of PD-L1 overexpression MC-38 cell line was similar to the above.

To knock out mouse RNF125 in MC-38 and H22 cells, small guide RNA (sgRNA) targeting RNF125 was designed and inserted into the LentiCrispr v2 vector to construct transfer plasmids. 293T cells were transfected with pMD2. G, psPAX2, and the corresponding transfer plasmid to produce lentivirus. A total of 10^8^ cells were infected with lentivirus at an MOI of 2.0 and selected with 4μg ml^-1^ puromycin for two weeks to ensure proper selection. The following sgRNA sequences were used:

sgRNA- 5′- CACCGTTGCGGGCACTCCCTCTGA-3′

control- 5′-CACCGCGCTTCCGCGGCCCGTTCAA-3′.

### Cell culture and transfection

Cultured HepG2, H22, MC-38, A549, and HEK293T cells in DMEM supplemented with 10% heat-inactivated fetal bovine serum (FBS, Invitrogen), 100 U/ml penicillin, and 100 mg/ml streptomycin. Using jetPRIME reagent for transient transfection according to the manufacturer’s instructions, all the cells tested were free of mycoplasma contamination.

### Western blotting and immunoprecipitation

Cell extracts were prepared, immunoprecipitated, and analyzed as previously described (17). In short, we collected cells into a modified RIPA buffer and treated them with ultrasound to prepare whole-cell lysates, loaded them into SDS-PAGE gels with about 60-80 μg proteins per lane and performed Western blot analysis. We used ultrasound to prepare whole cell lysates in NP40 buffer (1% NP40, 150 mM NaCl and 40 mM Tris at pH 7.5) for Co-immunoprecipitation. Then we cultured the lysate supernatant with primary antibody for 3 hours, then with protein G-Agarose (Roche) for 1 hour, and finally extensively washed with NP40 buffer.

### Ubiquitylation assays

Ubiquitylation assay was previously described (18). Briefly, Flag-tagged PD-L1, HA-tagged RNF125, and Myc-tagged ubiquitin were overexpressed in HEK293T cells for 24 hours. Four hours prior to collection, MG132 was added. For total cell lysis, Drosophila embryos or cells were lysed in a denaturing buffer (1% SDS, 50 mM Tris at pH 7.5, 0.5 mM EDTA, 1 mM DTT) and boiled for 10 minutes to disrupt protein-protein interactions. Lysates were produced by adding the protease inhibitors and lysis buffer described above. Ubiquitination was assessed by IP with an antibody against the Flag tag, followed by western blotting with an anti-Myc antibody.

### Immunofluorescence assay

We fixed the growing cells grown on poly-L-lysine-coated coverslips in 4% PFA for 10 minutes and then washed them with PBS 3 times. A PD-L1 antibody (1:100) and Alexa Fluor 647-labeled donkey anti-rabbit IgG (H+L) (1:100) were used for immunofluorescence staining. We were using DAPI to perform Nuclear DNA staining and using Nikon A1 Elements software (Version 4.20) and Nikon A1 confocal microscope to acquire Fluorescent images, and using Volocity (Version 6.1.1; PerkinElmer) to process these images.

### Immunohistochemical assay

The tumor tissues of mice were fixed with 4% paraformaldehyde and treated as follows. Deparaffinzing and rehydrating the paraffin section, antigen retrieval, blocking endogenous peroxidase activity, serum sealing, primary antibody incubation: the sealing solution was gently removed, the primary antibody prepared with PBS (PH7.4) in a certain proportion was added to the sections (CD4, CD8, F4/80), and the sections were placed flat in a wet box and incubated overnight at 4°C. Secondary antibody incubation, DAB chromogenic reaction, nucleus counterstaining, dehydration and mounting. Visualized staining of tissue under a microscope followed by image acquisition and analysis. The nucleus of hematoxylin stained was blue, and the positive expression of DAB is brownish yellow.

### T cell-mediated tumor cell killing assay

OVA-specific CD8 T cells were isolated from the spleens of OVA-specific T cell receptor transgenic (OT-I) mice using the Lymphocyte Separation Medium and EasySep™ Mouse CD8+ T Cell Isolation Kit. Naïve OVA-specific CD8+ mouse T cells were stimulated using Dynabeads Mouse T-Activator CD3/CD28 and expanded with mouse IL-2 cytokines (30 U/ml). MC-38-OVA cells were seeded into 12-well plates at a cell-dependent concentration. After 24 h, activated OVA-specific CD8+ T cells were co-cultured with adherent MC-38-OVA cells for 24 h and 48h at a ratio of 3:1. After 24h and 48h of incubation, T cells and cell debris were removed. For fluorescence-activated cell sorting (FACS) analysis, T cells were collected, MC-38-OVA cells were harvested and labeled with annexin-V and PI, and early (Annexin-V positive; PI negative) and late apoptosis (Annexin-V positive; PI positive) were detected using the Annexin V-FITC Apoptosis Detection Kit.

### Animal experiments

C57BL/6N Wild Type (WT) mice (8 to 10 weeks old) were purchased from SPF Biotechnology (Beijing, China). All the mice were group-housed conventionally on a 12-h light/dark cycle for three days before any experiments. All the animal experiments were performed at the AMMS Animal Center (Beijing, China) and were approved by the Institutional Animal Care and Use Committee. MC-38 or H22 tumor cells (2 × 10^6^) were injected into the abdominal mammary fat pad of 8-wk-old WT mice to build the syngeneic model. Tumor growth was monitored using caliper measurements. Excised tumors were weighed, and portions were frozen in liquid nitrogen or fixed in 4% (vol/vol) paraformaldehyde for further study.

### Statistical analysis

The correlation between gene expression and immune cell infiltration was analyzed using the TISIDB (http://cis.hku.hk/TISIDB/). Kaplan–Meier Plotter (https://kmplot.com/analysis/) was used to analyze the association of RNF125 with overall survival (OS) and relapse-free survival (RFS) in patients with multiple cancers derived from the TCGA database. The RNF125 gene levels were divided into high and low expression groups according to the cut-off values of the algorithm (19). Expression of the RNF125 gene in cancer tissues and normal tissues, and the relationship between RNF125 expression level and tumor stage were analyzed by UALCAN (http://ualcan.path.uab.edu/analysis.html) website. Statistical analysis was performed using SPSS 23.0 (SPSS Inc., Chicago, IL). All statistical tests were two-sided tests, and P values < 0.01 and 0.05 were deemed statistically significant. In all the figures and tables, * indicates *P <*0.05, ** indicates *P <*0.01, *** indicates *P <*0.001 **** indicates *P <*0.0001.

## Results

### RNF125 interacts with PD-L1 and regulates its expression

**Figure 1:**
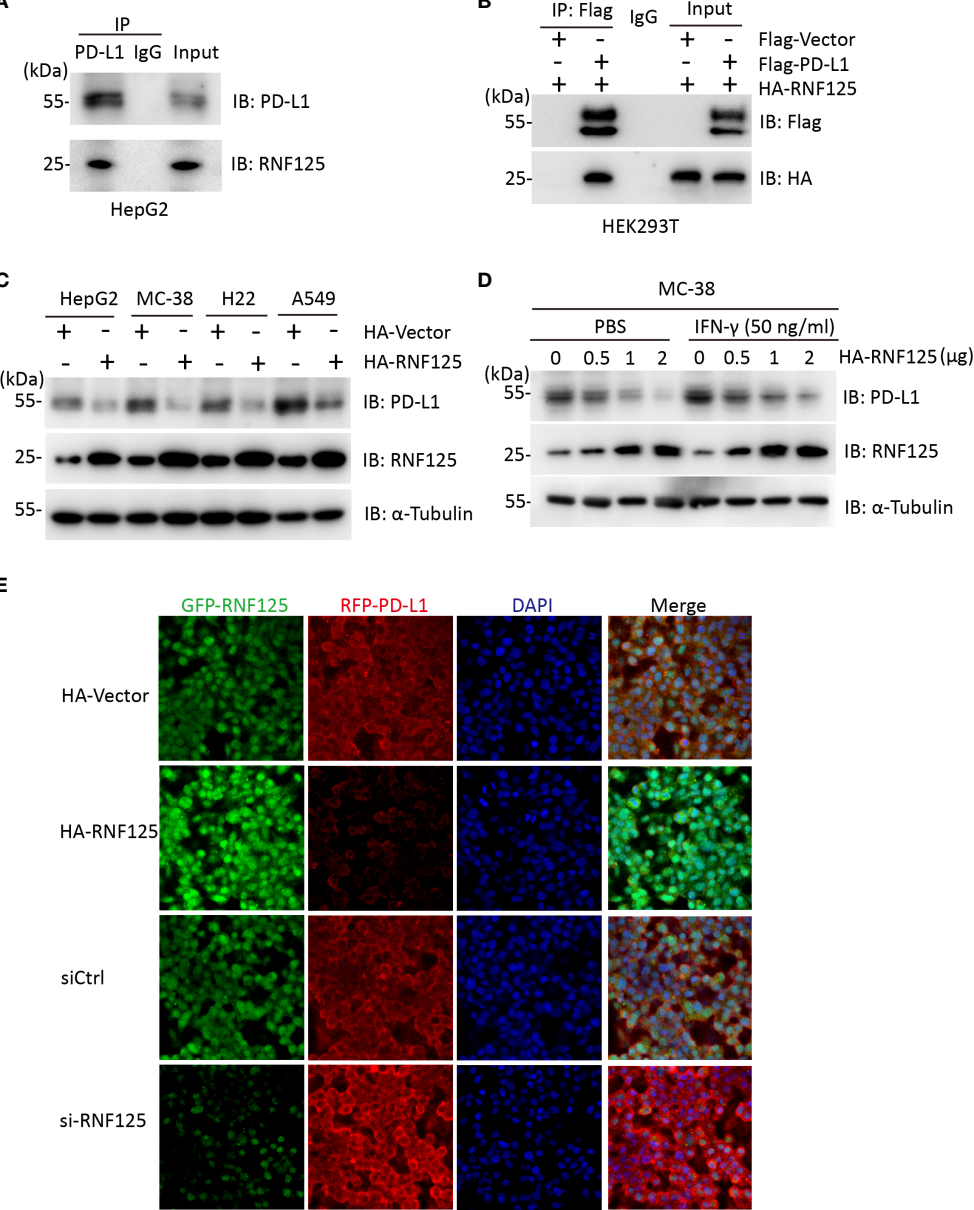
RNF125 interacts with PD-L1 and regulates its protein level **(A)** Co-immunoprecipitation analysis of HepG2 cells with an anti-RNF125 or anti-PD-L1 antibody. **(B)** Co-immunoprecipitation of HEK293T cells transfected with HA-RNF125 and Flag-PD-L1 or Flag-Vector. Anti-Flag or IgG agarose immunoprecipitates were analyzed using immunoblotting with an anti-Flag antibody or anti-HA antibody. **(C)** HA-RNF125 was transfected into HepG2, MC-38, H22, and A549 cells. Whole-cell lysates were used for western blotting with anti-PD-L1 or anti-RNF125 and α-tubulin antibodies. **(D)** Increasing amounts of HA-RNF125 were transfected into MC-38 cell line in the control group, the experimental group was treated with IFN-γ under the same conditions, Whole-cell lysates were used for western blotting with anti-PD-L1 or anti-RNF125 and anti-α-tubulin antibodies. **(E)** Representative confocal immunofluorescence images of PD-L1 in HepG2 cells transfected with HA-Vector, HA-RNF125, siControl (siCtrl) or si-RNF125 RNAi oligos. Red represents PD-L1, green represents RNF125 and blue represents the nuclei.

We first explored the interaction between RNF125 and PD-L1. To this end, we used HepG2 cells for Co-Immunocoprecipitation (Co-IP) experiment. Endogenous RNF125 could be detected in protein A/G beads which captured the PD-L1 immuno-complex (**Figure 1A**). Similarly, HA-RNF125 and Flag-PD-L1 expression plasmids were co-transfected into HEK293T cells, and Co-IP was performed. HA-RNF125 was detected in the anti-Flag immunoprecipitate from cells cotransfected with Flag-PD-L1 but not with Flag-vector (**Figure 1B**).

Next, we examined whether PD-L1 was a candidate substrate of RNF125. RNF125 was overexpressed in HepG2, MC-38, H22 and A549 cell lines. The results showed a significant decrease in PD-L1 protein levels when RNF125 levels were increased (**Figure 1C**). We also found that PD-L1 level was decreased when RNF125 level was increased with or without treatment of IFN-γ in MC-38 cells (**Figure 1D**). Furthermore, confocal microscopy showed that the staining intensity of PD-L1 was significantly decreased following RNF125 overexpression, whereas RNF125 knockdown led to increased PD-L1 expression (**Figure 1F**). These results indicated that RNF125 interacts with PD-L1 and regulates PD-L1 expression.

### RNF125 induces K48-linked ubiquitination of PD-L1 for degradation

**Figure 2:**
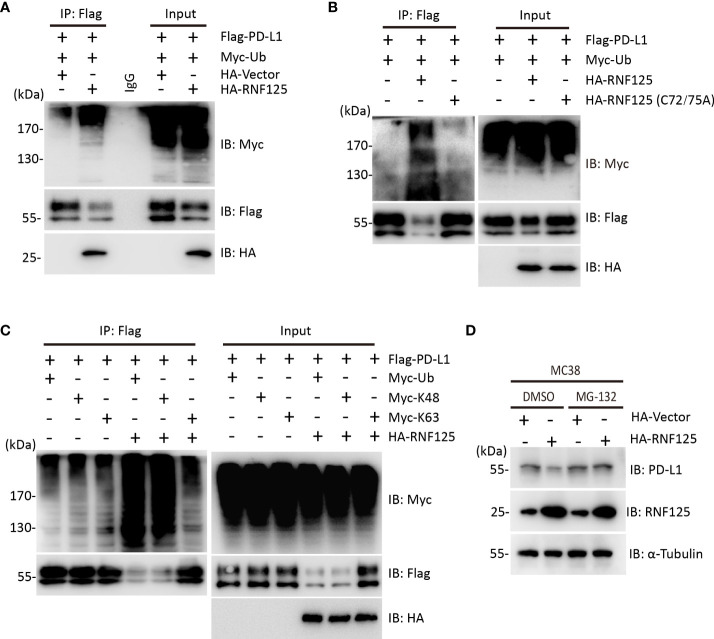
RNF125 induces K48-linked ubiquitination of PD-L1 for degradation. **(A)** Flag-RNF125, Myc-ubiquitin (Myc-Ub) and HA-PD-L1 were cotransfected into HEK293T cells. Anti-Flag immunoprecipitates were analyzed using immunoblotting with an anti-Myc or anti-Flag antibody. The levels of the transfected proteins were analyzed using immunoblotting with an anti-HA, anti-Myc, or anti-Flag antibody. **(B)** Flag-RNF125 or Flag-RNF125 C72/75A expression plasmids were cotransfected into HEK293T cells with the HA-PD-L1 expression plasmid and Myc-Ub. Anti-Flag immunoprecipitates were analyzed using immunoblotting with an anti-Myc or anti-Flag antibody. The levels of the transfected proteins were analyzed using immunoblotting with an anti-HA, anti-Myc, or anti-Flag antibody. **(C)** Flag-RNF125 and HA-PD-L1 were cotransfected with Myc-Ub, Myc-Ub (K48) or Myc-Ub (K63) into HEK293T cells. Anti-Flag immunoprecipitates were analyzed using immunoblotting with an anti-Myc or anti-Flag antibody. The levels of the transfected proteins were analyzed using immunoblotting with an anti-HA, anti-Myc, or anti-Flag antibody. **(D)** HA-vector or HA-RNF125 was transfected into MC-38 cell line in the presence of DMSO or MG132 (10 µM). After 24 h, RNF125, PD-L1 expression was analyzed by WB using anti-PD-L1 or anti-RNF125 antibodies.

To test whether RNF125 was the E3 ubiquitin ligase for PD-L1, ubiquitination assay was performed. HEK293T cells were cotransfected with Flag-PD-L1 and Myc-Ubiquitin in the presence or absence of HA-RNF125. The level of PD-L1 ubiquitination in the presence of HA-RNF125 was significantly increased compared with that in the Flag-Vector group (**Figure 2A**). Notably, RNF125 enhanced PD-L1 ubiquitination specifically *via* E3 ligase activity because the RNF125 C72/75A mutant (without E3 ligase activity) displayed compromised PD-L1 ubiquitination activity (**Figure 2B**). We then co-expressed Myc-tagged ubiquitin mutants with Flag-PD-L1 in the absence or presence of RNF125 to characterize the ubiquitin chains on PD-L1. The results showed that RNF125 catalyzed the ubiquitination of PD-L1 by K48-specific ubiquitination, a form of ubiquitin chain targeting proteins for proteasomal degradation (**Figure 2C**). Next, we treated cells with the proteasome inhibitor MG132 to examine the effect of RNF125 on PD-L1 expression. RNF125 failed to downregulate PD-L1 expression following MG132 treatment (**Figure 2D**). Together, these results indicated that RNF125 induces K48-linked ubiquitination of PD-L1 for proteasomal degradation.

### RNF125 inhibits tumor growth and promotes tumor immunity

**Figure 3:**
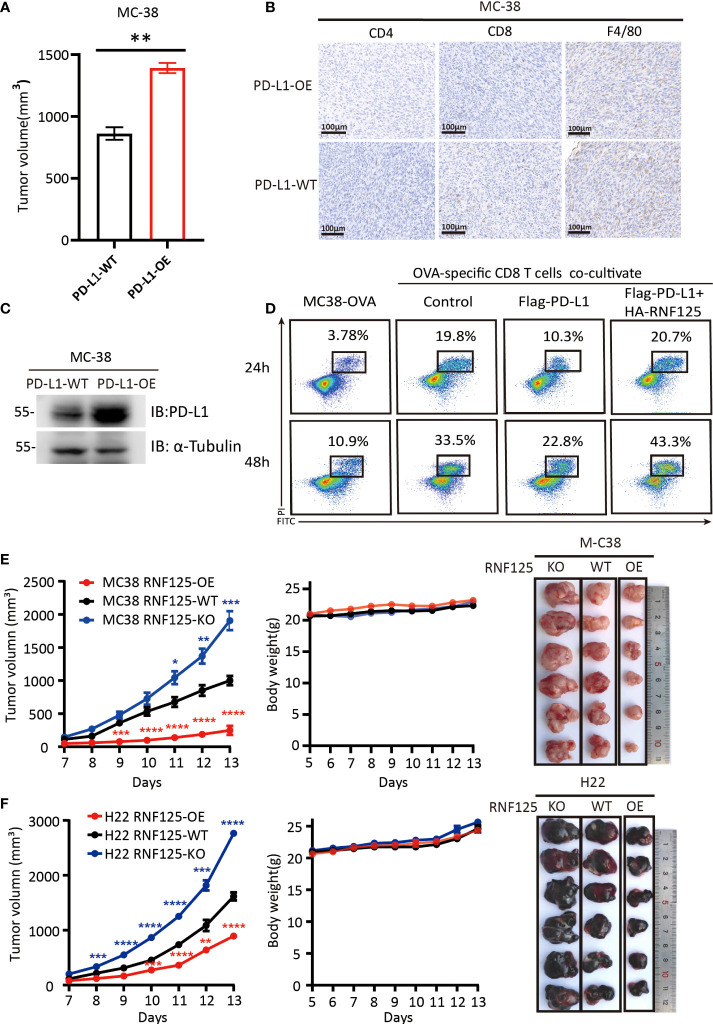
RNF125 inhibits tumor growth and promotes tumor immunity **(A–C)** Correlation analysis between PD-L1 protein level and immune infiltration **(A)** The volume of tumors were compared between MC-38 tumor with PD-L1 overexpression (PD-L1-OE) and it’s parental tumor (PD-L1-WT) (n=3), **(B)** PD-L1 immune infiltration level of these two tumors were analyzed by IHC using anti-CD4, anti-CD8 and anti-F4/80 antibodies. **(C)** PD-L1 protein level of these two tumors were analyzed by WB using anti-PD-L1 and anti-α-tubulin. **(D)** Representative images of FACS analysis of OVA-specific CD8 T cell-mediated elimination of MC-38-OVA cells, as determined by annexin V-FITC and propidium iodide (PI) double labeling. **(E, F)** Tumor volume, body weight and representative images of the syngeneic tumors derived from MC-38 **(E)** or H22 **(F)** RNF125-KO, RNF125-WT and RNF125-OE cell line syngeneic mouse models. (n=6). (* indicates *P* <0.05, ** indicates *P* <0.01, and *** indicates *P* <0.001, **** indicates *P* <0.0001).

To verify the functional importance of RNF125 in cancer immune escape, we first constructed a PD-L1-overexpressing (PD-L1-OE) MC-38 cell line and implanted subcutaneously in C57BL/6N mice. It was found that mice bearing PD-L1-OE MC-38 tumors grow faster compared with the parental MC-38 cell line (PD-L1-WT) (**Figure 3A**). Immunohistochemical staining showed that the CD4^+^, CD8^+^T cell, and macrophage infiltration levels of PD-L1-OE tumor were lower than that of the PD-L1-WT parental tumor (**Figure 3B**). Western blot assay also confirmed that PD-L1-OE tumor expressed significantly higher levels of PD-L1 than PD-L1-WT (**Figure 3C**). This proves that PD-L1 level in tumor was negatively correlated with immune infiltration level. Then, the effect of RNF125 on T cell-mediated killing assays was performed. The *in vitro* results indicated that PD-L1 expression rendered tumor cells more resistant to T cell-mediated cytolysis, whereas RNF125 overexpression resensitized cytolysis killing of T cells (**Figure 3D**).

**Figure 4:**
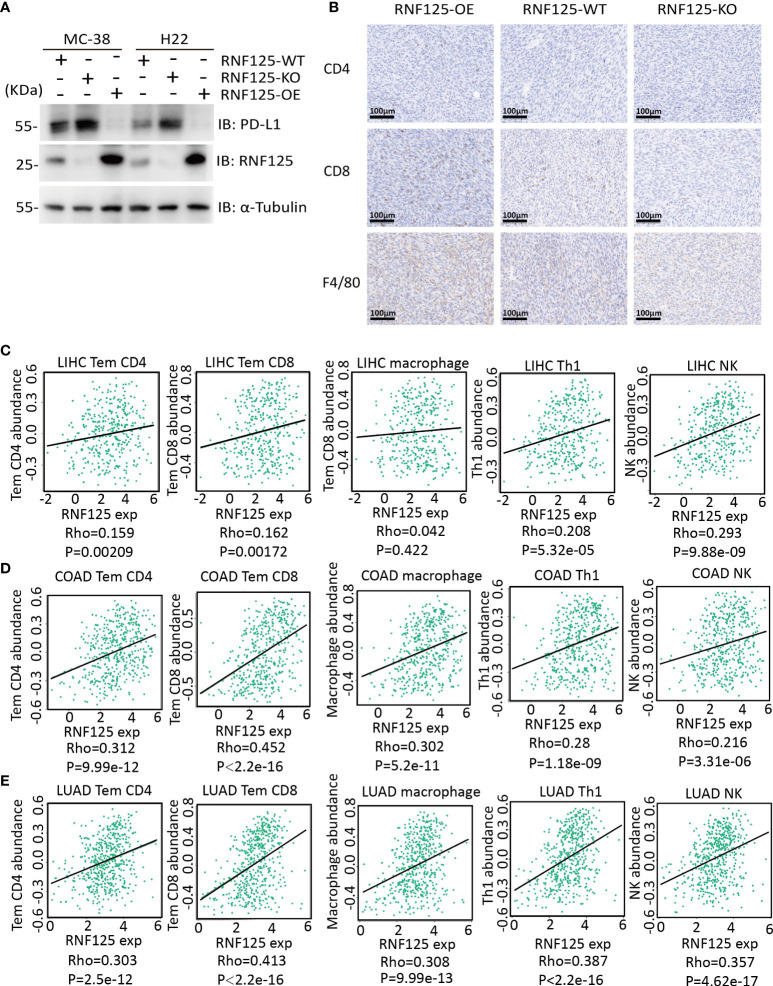
High RNF125 levels are associated with increased immune cell infiltration **(A)** RNF125, PD-L1, and α-tubulin expression in different tumor samples were analyzed by WB using anti-PD-L1 or anti-RNF125 and anti-α-tubulin antibodies. **(B)** CD4, CD8, and F4/80 positive cell levels in different tumor samples were analyzed by IHC using anti-CD4, anti-CD8, and anti-F4/80 antibodies. **(C)** Correlation analysis between RNF125 expression levels and Tem CD4, Tem CD8, macrophage, Th1 cell, and NK cell infiltration levels in LIHC using “TISIDB” website. **(D)** Correlation analysis between RNF125 expression levels and Tem CD4, Tem CD8, macrophage, Th1 cell, and NK cell infiltration levels in COAD using “TISIDB” website. **(E)** Correlation analysis between RNF125 expression levels and Tem CD4, Tem CD8, macrophage, Th1 cell, and NK cell infiltration levels in LUAD using “TISIDB” website.

Next, we established of subcutaneous transplanted tumor model in C57BL/6N mice using H22 and MC-38 cell lines with RNF125 knockout (RNF125-KO) or RNF125 overexpression (RNF125-OE). Notably, MC-38 and H22 cell lines with RNF125 knockout exhibited a higher PD-L1 level and significantly faster tumor growth than their parental cell lines. In contrast, overexpression of RNF125 in MC-38 and H22 cells had the opposite effect, resulting in lower PD-L1 levels and delayed tumor growth compared with parental cell lines (**Figures 3E, F**, **4A**). Additionally, contrary to the tumor results of RNF125-OE, reduced expression of CD4, CD8, and F4/80 in RNF125 KO tumors was observed (**Figure 4B**).

In order to verify the relationship between RNF125 and immune infiltration in Colon Adenocarcinoma (COAD), Liver Hepatocellular Carcinoma (LIHC), and Lung Adenocarcinoma (LUAD), we used “TISIDB” website, an integrated repository portal for tumor-immune system interactions, for in-depth immune infiltration analysis.

We found a positive correlation between RNF125 gene expression level and TEM CD4 (Effector memory CD4 T cell), TEM CD8 (Effector memory CD8 T cell), macrophage, type 1 T helper cell (Th1) and natural killer cell (NK) in LIHC, COAD, and LUAD (**Figures 4C–E**). Such a relationship also exists in rectum adenocarcinoma (READ) and lung squamous cell carcinoma (LUSC), (**Table 1**). These results indicated that RNF125 could participate in the tumor immunity by promoting PD-L1 ubiquitination and degradation.

**Table 1:** Correlation between the RNF125 gene levels and immune infiltration level using “TISIDB”.

Tumor type	TEM CD4		TEM CD8		Macrophage		Th1		NK	
	R value	P value	R value	P value	R value	P value	R value	P value	R value	P value
COAD	0.312	***	0.452	***	0.302	***	0.28	***	0.216	***
READ	0.298	***	0.273	***	0.249	**	0.168	*	0.104	0.18
LUAD	0.303	***	0.413	***	0.308	***	0.387	***	0.357	***
LUSC	0.35	***	0.404	***	0.35	***	0.414	***	0.451	***
LIHC	0.159	**	0.162	**	0.042	0.422	0.208	***	0.293	***

Effector memory CD4 T cell (TEM_CD4), Effector memory CD8 T cell (TEM_CD8), Type 1 T helper cell (Th1), Natural killer cell (NK).

* indicates P <0.05, ** indicates P <0.01, and *** indicates P <0.001.

COAD, Colon Adenocarcinoma; READ, Rectum adenocarcinoma; LUAD, Lung Adenocarcinoma; LUSC, lung squamous cell carcinoma; LIHC, Liver Hepatocellular Carcinoma.

### RNF125 is decreased in the process of tumor occurrence and progression

**Figure 5:**
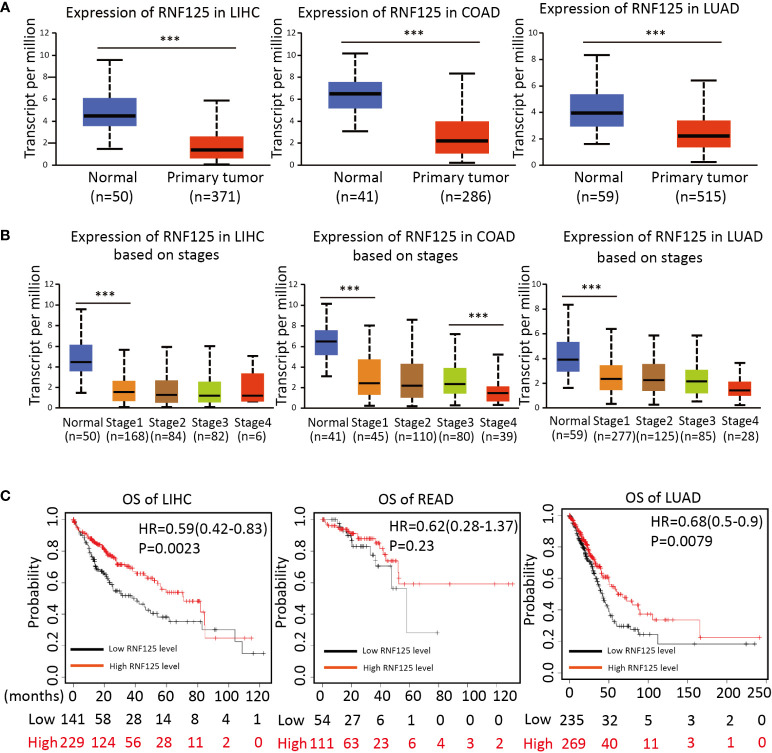
Expression of RNF125 in tumor tissues and its relationship with clinical outcomes **(A)** The difference of RNF125 expression levels between normal and tumor tissues of LIHC, COAD, and LUAD were analyzed. **(B)** Analysis of the expression of RNF125 levels in different clinical stages in LIHC, COAD, and LUAD. **(C)** Kaplan-Meier was used to evaluate the relationship between RNF125 expression and overall survival in LIHC, READ, and LUAD tumors. *** indicates P <0.001.

To investigate the potential clinical role of RNF125 in cancers, we then used ”UANCAN” website to analyze the expression of RNF125 in tumors and normal tissues. The results showed that RNF125 expression was lower in COAD, LIHC and LUAD tumor tissues than in normal tissues (**Figure 5A**). Further analysis showed that the expression of the RNF125 gene was negatively correlated with clinical staging of tumor (**Figure 5B**). Thus, the analysis showed that the level of RNF125 may be decreasing in the process of tumor occurrence and progression.

### High RNF125 expression is associated with a better clinical outcome

Using Kaplan–Meier survival analysis curves, which divided the RNF125 into high and low expression groups according to cutoff values, we next assessed the relationships between RNF125 and the clinical outcome of cancer. The results showed that the high level of RNF125 was associated with better OS in LIHC and LUAD. In addition, although there was no significant difference between RNF125 and OS in READ (p=0.23), the median survival time of patients with a high level of RNF125 was also greater than patients with low RNF125 level (43.8 vs 36.53 months) (**Figure 5C**).

We next used HCC as an example and conducted OS and RFS subgroup analyses on the clinicopathological features of HCC patients, including tumor stage, tumor grade, vascular infiltration, sex, race, sorafenib treatment, alcohol intake, and hepatitis virus. High RNF125 expression was associated with better RFS for tumor stage 1, grade 2 disease, female gender, white patients, Asian patients, sorafenib treatment, no alcohol intake, and hepatitis virus-free status, however, tumor stage 2, grade 1 disease, vascular infiltration, and female patients were not associated with OS (**Tables 2**, **3**). These findings above suggested that part of patients were likely to benefit from high RNF125 expression.

**Table 2:** OS of HCC at different RNF125 levels.

Clinicopathological index	Subgroup	Cut off	Grouping (cases)			Median OS (months)		HR value	P value
			LEG	HEG	LEG		HEG		
	Entire queue	182	122	242	31		70.5	0.57 (0.4-0.81)	1.50E-03
Stage	Stage 1	367	82	88	56.5		84.4	0.46 (0.24-0.86)	0.013
	Stage 2	164	27	56	28.3		13.8	0.5 (0.23-1.08)	0.0724
	Stage 3	323	48	35	14		40.3	0.49 (0.26-0.92)	0.024
Grade	Grade 1	313	17	38	10		47.4	0.54 (0.2-1.43)	0.21
	Grade 2	195	50	124	25.6		81.9	0.51 (0.3-0.86)	0.011
	Grade 3	358	78	40	13.7		22	0.45 (0.22-0.94)	0.029
Vascular invasion	None	171	54	149	37.8		81.9	0.61 (0.36-1.05)	0.072
	Micro	110	21	69	82.9		108.6	0.57 (0.25-1.32)	0.1831
Gender	Male	367	139	107	42.4		84.7	0.43 (0.27-0.71)	6.00E-04
	Female	184	42	76	37.8		56.5	0.74(0.42-1.3)	0.2917
Race	White	207	54	127	37.8		54.1	0.67(0.42-1.07)	0.0905
	Asian	341	91	64	NA		NA	0.26(0.12-0.56)	2.00E-04
Sorafenib treatment		196	9	20	21.1		45.7	0.17(0.05-0.62)	0.0002
Alcohol consumption	Yes	362	60	55	41		70.5	0.4(0.21-0.78)	0.0052
	None	171	71	131	30		71	0.55(0.35-0.87)	0.0091
Hepatitis virus	Yes	174	49	101	14.2		54.1	0.49(0.25-0.93)	0.0249
	None	312	85	82	21.4		70.5	0.46(0.29-0.74)	1.00E-03

Micro, microvascular invasion.

LEG: The RNF125 low expression group distinguished by cutoff values.

HEG: The RNF125 high expression group distinguished by cutoff values.

**Table 3:** RFS of HCC at different RNF125 levels.

Clinicopathological index	Subgroup	Cut off value	Grouping (cases)		Median RFS (months)		HR value	P value
			LEG	HEG	LEG	HEG		
	Entire queue	599	229	87	21.93	42.87	0.62(0.42-0.92)	0.0154
Stage	Stage 1	155	37	116	30.1	56.67	0.51(0.29-0.91)	0.021
	Stage 2	568	55	20	6.97	8.4	0.65(0.28-1.51)	0.3116
	Stage 3	196	31	39	7.3	14.33	0.63(0.35-1.16)	0.1326
Grade	Grade 1	736	29	16	8.83	18.87	0.46(0.15-1.4)	0.16
	Grade 2	321	66	83	17.9	30.4	0.56(0.34-0.91)	0.0172
	Grade 3	436	79	28	25.87	10.4	1.47(0.84-2.6)	0.1776
Vascular invasion	None	200	51	124	37.23	42.63	0.75(0.45-1.25)	0.2672
	Micro	519	60	22	13.33	37.67	0.46(0.02-1.05)	0.0593
Gender	Male	590	155	55	21.87	42.63	0.66(0.41-1.05)	0.0804
	Female	147	29	77	11.83	55.87	0.52(0.28-0.97)	0.036
Race	White	674	108	39	16.3	36.1	0.54(0.3-0.96)	0.0325
	Asian	158	52	93	15.97	54.33	0.52(0.31-0.87)	0.0119
Sorafenib treatment		746	14	8	6.33	16.6	0.33(0.12-0.97)	0.036
Alcohol consumption	Yes	259	40	59	13.33	36.1	0.7(0.39-1.26)	0.2321
	None	171	65	118	15.17	42.63	0.6(0.38-0.94)	0.0231
Hepatitis virus	Yes	405	87	52	25.13	42.63	0.68(0.4-1.16)	0.1598
	None	124	35	108	8.7	34.4	0.51(0.29-0.89)	0.0166

## Discussion

Currently, cancer immunotherapy mainly focuses on immune inhibitory checkpoints, including CTLA-4, PD-L1, and PD-1 (20). Monoclonal antibodies blocking these immune checkpoints lead to unleashed anti-tumor immunity and durable clinical responses in a subset of patients with advanced cancers (21). Failing to induce clinical responses in most patients re-emphasizes the need to address the key molecular determinants responsible for immune dysfunction in the tumor micro-environment (22). Our study reveals that RNF125 can interact with PD-L1 and negatively regulate PD-L1 expression through K48-linked polyubiquitination. Notably, compared with RNF125-WT tumors, RNF125-KO tumors exhibited higher PD-L1 expression levels and progressed significantly faster. In contrast, RNF125-OE tumors exhibited opposite characteristics with lower PD-L1 expression and slower progression. Consistent with these findings, immune infiltration analysis suggested that the expression level of RNF125 showed a positive correlation with CD4+, CD8+T cells, and macrophages. Hence, enhanced RNF125 expression or E3 ligase activity may exert tumor-suppressive effects in cancer cells. Additionally, RNF125 expression was significantly down-regulated in multiple human cancer tissues, and patients with higher RNF125 expression had better clinical outcomes. Subgroup analysis of HCC suggests that in some clinicopathological states, such as in patients receiving sorafenib treatment or in patients with alcoholism or hepatitis virus infection, biopsy monitoring of RNF125 levels may benefit the prediction of RFS and OS and guide clinical treatment, but requires further clinical studies to demonstrate.

Ubiquitination modification is a rapidly inducible and reversible process known to target damaged proteins for degradation and modulate the localization, function and interactions of target proteins (23). The modification of proteins by ubiquitination requires the sequential activity of ubiquitin-activating enzyme E1, ubiquitin-conjugating enzyme E2, and ubiquitin ligases E3. These E3s can be recognized by the presence of the best-characterized E2 interacting domains (24). A previous study showed that RNF125 was identified from a T cell surface activation marker screen and belonged to the subfamily of RING ubiquitin ligases (25). Previously study indicated that RNF125 was the first E3 ubiquitin ligase for T cell activation (26). RNF125 was also found to ubiquitinate RIG-I and P53, providing a potential role for RNF125 in tumorigenesis and innate immunity (27, 28). Moreover, RNF125 has been shown to inhibit HIV replication and cytokine production. Additionally, RNF125 also regulates IL-36R that contributes to cell proliferation and inflammatory responses (29). Our results showed that RNF125 was down-regulated in various cancers. RNF125/PD-L1 may contribute to the immune evasion of a subset of tumors. We observed that high RNF125 expression in multiple tumors was associated with a better clinical outcome, and the complex and multifaceted roles of RNF125 in multiple pathways warrant further investigation.

## Data availability statement

The original contributions presented in the study are included in the article. Further inquiries can be directed to the corresponding authors.

## Ethics statement

The animal study was reviewed and approved by Affiliated Tumor Hospital of Guangxi Medical University (Ethics number, LW2022095).

## Author contributions

MW, YM, JLL, and JZ performed the experiments and wrote the manuscript. HL, YX, YP, and ZT analyzed and interpreted the data. TW, XY, LH, and XS performed part of cell experiments. JFL and LZ performed part of animal experiments. HZ, CW, QX, MM, and FW designed and guided the project. All authors contributed to the article and approved the submitted version.

## Funding

This work was supported by grants from the National Natural Science Foundation of China (NSFC) (No. 81860502, No. 81301871) and Key Laboratory of Early Prevention and Treatment for Regional High Frequency Tumor, Ministry of Education (GKE2019-06, GKE-ZZ202004, GKE-ZZ202129). Key project of Guangxi Science and Technology Department (Gui Ke AB16380242).

## Conflict of interest

The authors declare that the research was conducted in the absence of any commercial or financial relationships that could be construed as a potential conflict of interest.

## Publisher’s note

All claims expressed in this article are solely those of the authors and do not necessarily represent those of their affiliated organizations, or those of the publisher, the editors and the reviewers. Any product that may be evaluated in this article, or claim that may be made by its manufacturer, is not guaranteed or endorsed by the publisher.
